# Sickle Cell Trait Presenting as Unilateral Proliferative Retinopathy and Macular Thinning in a Pregnant Woman

**DOI:** 10.1155/2021/5988889

**Published:** 2021-12-11

**Authors:** Sepideh Ghods, Elias Khalili Pour, Hooshang Faghihi, Golnaz Gharehbaghi, Ahmad Mirshahi, Fariba Ghassemi, Bahman Inanloo, Hamid Riazi-Esfahani

**Affiliations:** ^1^Eye Research Center, Farabi Eye Hospital, Tehran University of Medical Sciences, Tehran, Iran; ^2^Aliasghar Clinical Research Development Center, Aliasghar Children Hospital, Iran University of Medical Sciences, Tehran, Iran

## Abstract

**Background:**

To report a case of a pregnant woman with sickle cell trait (SCT) who presented with unilateral proliferative sickle cell retinopathy. *Case Presentation*. A 26-year-old otherwise healthy pregnant woman presented with the complaint of visual loss in her left eye. The funduscopic examination showed vitreous hemorrhage, sea fan neovascularization, and pale optic disc. Optical coherence tomography revealed macular inner retinal layer thinning and foveal splaying (saucerization of the foveal pit). The investigations, including hemoglobin electrophoresis, verified the diagnosis of sickle cell trait. Blood pressure, fasting blood glucose tests, and tuberculin skin tests were normal. We treated the patient by peripheral retinal photocoagulation over the area of nonperfusion.

**Conclusion:**

Even though the sickle cell trait is generally considered as a milder form of sickle cell disease without severe retinal manifestations, pregnancy should be considered as a trigger that can induce proliferative changes and foveal splaying in this group of patients.

## 1. Introduction

Normal adult hemoglobin (hemoglobin A) is composed of two alpha and two beta subunits. If point mutation in the beta subunit allele results in substitution of glutamic acid by valine or lysine at position 6, hemoglobin S (HbS) and C (HbC) will be formed, respectively. Sickle cell disease (SCD) is the homozygote state of the HbS allele (SS), and sickle cell trait (SCT) occurs when an individual has only one HbS allele [1, 2].

SCD is associated with many systemic and ocular complications, which have been mostly attributed to vaso-occlusive events. Abnormal hemoglobin in SCD can be polymerized and creates sickle-shaped red cells which result in vascular obstruction and ischemia. The sickling process may be triggered by infections, dehydration, and acidosis [1, 3].

Sickle cell retinopathy has two stages: nonproliferative and proliferative sickle cell retinopathy (P-SCR) [3–5]. P-SCR occurs due to occlusion of the peripheral arterioles, at branching points by sickled erythrocytes. It has sequential stages described by Goldberg and consists of retinal arteriolar obstruction and peripheral retinal ischemia, formation of peripheral vascular anastomosis, sea fan neovascularization at the border of the perfused and ischemic retina, occurrence of vitreous hemorrhage, and tractional retinal detachment, respectively [6]. The macula can also be impaired in the course of sickle cell retinopathy [7].

SCT was expected to be a benign condition for a long time with some protection against malaria [8]. However, numerous case reports have demonstrated a variety of complications in patients with SCT which previously were associated with SCD, like splenic infarction, hemolytic anemia, necrosis of the femoral head, renal cortical infarcts, venous thromboembolic disorders, and some pregnancy-related complication with no consequences on average life span [9–11]. Furthermore, nonproliferative retinal manifestations, as well as retinal artery occlusion, chorioretinal infarction, and angioid streaks, have been reported in patients with SCT. In the vast majority of these reports, there were concomitant systemic diseases [9].

Herein, we report a 26-year-old pregnant woman with SCT who had presented with proliferative retinopathy in her left eye and successfully treated with peripheral laser ablation.

## 2. Case Presentation

A 26-year-old woman was presented with reduced vision in her left eye two weeks ago. She was 6 months pregnant, and all perinatal evaluations were normal. At presentation, the best-corrected visual acuity was 10/10 in the right eye and counting finger at 2 meters in the left eye. Her past medical and ocular history was unremarkable.

**Figure 1:**
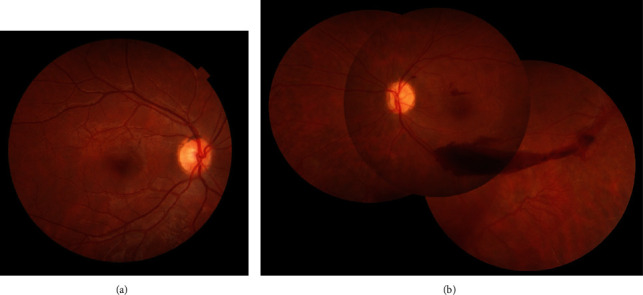
Fundus photograph of the right eye (a) revealed normal fundus appearance. Montage fundus photograph of the left eye (b) revealed vitreous hemorrhage, moderately pale optic disc, macular hemorrhage, extensive peripheral retinal avascular area, sea fan neovascularization, and small fibrotic tissue in the temporal midperipheral retina.

Ocular examination was completely normal on the right eye. Anterior segment examination of the left eye was unremarkable. Funduscopic examination of the left eye revealed vitreous hemorrhage, moderately pale optic disc, macular hemorrhage, extensive peripheral retinal avascular area, sea fan neovascularization, and small fibrotic tissue in the temporal midperipheral retina (Figure 1).

**Figure 2:**
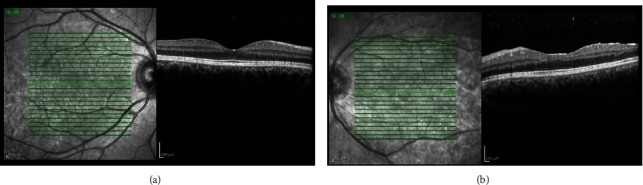
Horizontal macular optical coherence tomography (OCT) of the right eye (a) revealed the normal macular structure and in the left eye (b) revealed inner retinal irregularity and thinning along with foveal splaying (saucerization of the foveal pit).

Macular optical coherence tomography (OCT) (Heidelberg Engineering GmbH, Heidelberg, Germany) revealed macular inner retinal irregularity and thinning along with foveal splaying (saucerization of the foveal pit) (Figure 2).

**Figure 3:**
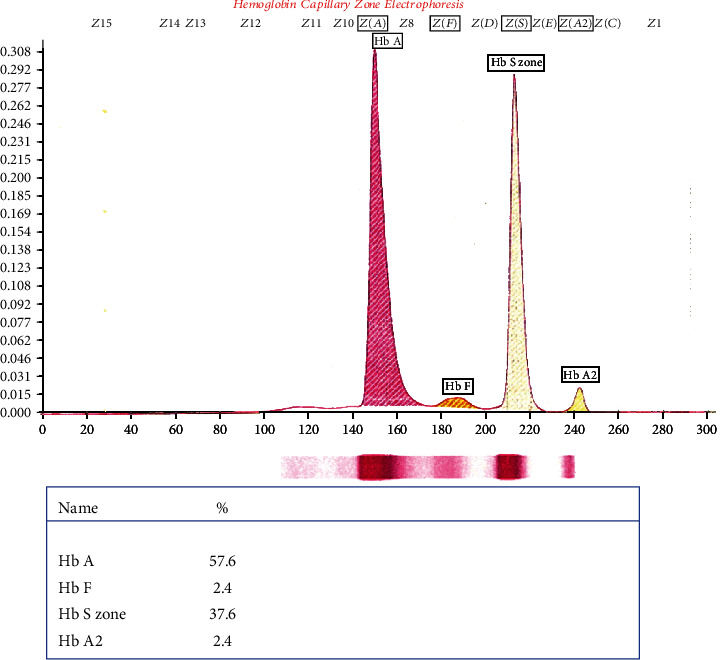
Hemoglobin electrophoresis showed the presence of 57.6% hemoglobin A (HbA), 37.6% hemoglobin S, 2.4% hemoglobin A2, and 2.4% hemoglobin F, which was compatible with the diagnosis of sickle cell trait.

Blood pressure was 110/70 mmHg. Fasting blood glucose and glucose tolerance test results were within normal range. Hemoglobin concentration was 14.0 g/dL. Other laboratory tests, including tuberculin skin test (0 mm induration), syphilis serology, human immunodeficiency virus (HIV) serology, liver function tests, renal function tests, and urine analysis, were all normal. Hemoglobin electrophoresis showed the presence of 57.6% hemoglobin A (HbA), 37.6% HbS, 2.4% HbA2, and 2.4% HbF, which was compatible with the diagnosis of sickle cell trait (Figure 3).

**Figure 4:**
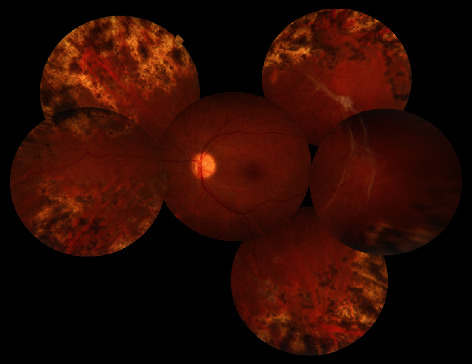
Montage fundus photograph of the left eye 6 months later revealed that vitreous and preretinal hemorrhages cleared, sea fan neovascularization partially regressed, and fibrotic membrane extended in the temporal midperipheral area.

**Figure 5:**
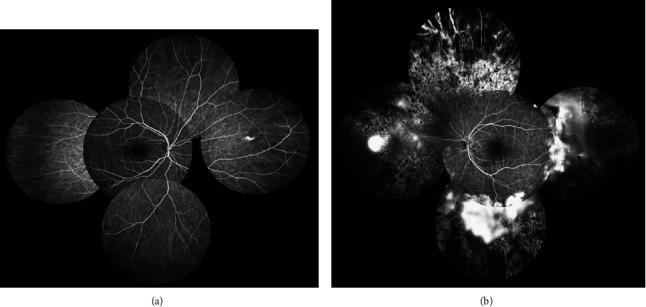
Montage midphase fluorescein angiography of the right eye (a) showed unremarkable findings but in the left eye (b) revealed residual peripheral avascular area, laser scars, and partially regressed neovascularizations corresponding to the leakage areas.

Argon laser photocoagulation was performed over the peripheral avascular area and the patient was referred to the gynecologist. The patient returned for follow-up 6 months later (3 months after delivery). Her visual acuity improved to 3/10. On examination, vitreous hemorrhage was cleared and sea fan neovascularization regressed clinically (Figure 4). Fluorescein angiography (Heidelberg Engineering GmbH, Heidelberg, Germany) showed normal foveal avascular zone size, residual peripheral avascular area, laser scars, and partially regressed neovascularization at the midperipheral retina (Figure 5). The patient was considered for additional targeted retinal photocoagulation over the nonregressed areas.

## 3. Discussion

Sickle cell hemoglobinopathies are among the most prevalent genetic disorders worldwide. Sickle cell trait (hemoglobin AS) is the most common genotype and has traditionally been considered a benign condition without severe ocular manifestations [9, 12]. However, several cases of ocular disease associated with sickle cell trait have been reported. There are a few other reports of proliferative retinopathy in SCT cases. In most of these studies, the SCT was accompanied by other systemic or ocular diseases such as diabetes, hypertension, sarcoidosis, tuberculosis, gestational diabetes, and ocular trauma [13–16]. Nagpal et al. suggest that the combination of systemic disease and SCT may be more pathogenic for retinopathy than any one of the conditions by itself. The presence of systemic disease creates unfavorable conditions that exacerbate the sickling process, resulting in ocular signs similar to sickle cell disease [13]. Therefore, the occurrence of retinopathy in sickle cell trait patients requires comprehensive medical assessments to rule out systemic diseases [12]. Here, we have reported a case of P-SCR in a pregnant woman with SCT.

Based on a study by Diaw et al., SCT could increase inflammatory and oxidative stress and lead to vascular endothelial cell impairment. Likewise, it could cause an increase in blood viscosity in these individuals [17]. On the other hand, previous studies revealed that pregnancy can increase the risk of some SCT-associated end-organ damages, particularly venous thromboembolism and chronic kidney disease. In addition, SCT women who conceived are at higher risk of some pregnancy-related problems such as bacteriuria, hypertensive disorders, abortion, and low birth weight [18]. The presence of proliferative retinopathy as well as maculopathy in an SCT pregnant woman without any systemic comorbidities has not been reported yet, and based on our knowledge, this is the first report of P-SCR in this group of patients.

The role of pregnancy in inducing proliferative retinopathy may be related to hypercoagulative state or hemodynamic changes. Marik and Plante reported a fourfold increase in thrombotic events in pregnant women [19]. Austin et al. showed that SCT patients on hormonal contraceptive medications had twelve times more chance of developing venous thromboembolism [20]. On the other hand, pregnancy leads to a considerable hemodynamic shift toward higher cardiac output and decreased peripheral vascular resistance. In turn, retinal vascular autoregulation causes retinal vascular constriction to adjust the blood volume entry to retinal vasculature [21]. All of these mechanisms could lead to more red cell sickling and retinal arteriolar obstruction in an SCT pregnant patient and, finally, development of P-SCR. Furthermore, higher hemoglobin levels and consequently higher blood viscosity in our patients (Hb = 14 g/dL) might intensify the occurrence of proliferative retinopathy. Condon and Serjeant previously reported a higher prevalence of proliferative retinopathy in patients with SC genotype with higher hemoglobin concentrations (Hb > 12.5 g/dL) [22].

Lim and Cao revealed inner macular layer thinning in SS, SC, and SThal genotypes which were more pronounced in SS, even in the asymptomatic patients. Macular thinning in their study was correlated with increasing age and stage of retinopathy [23].

Macular inner retina thinning which is called foveal splaying (saucerization of the foveal pit) was obvious in the left eye of the patient. Recently, Fares et al. suggested an independent relationship between macular thinning and retinopathy grading in patients with sickle cell retinopathy; they showed that foveal splaying was significantly more in eyes with P-SCR [24]. The fact that the temporal macular region and the retinal periphery are both fed by small-caliber terminal arterioles may explain close relationship of maculopathy and retinopathy in SCD patients; hence, vascular blockage can readily harm both areas. This finding is consistent with previous researches indicating that a greater prevalence of vaso-occlusive crises in HbSS patients may result in recurrent ischemic events in the eyes, resulting in severe macular thinning [25]. Therefore, the presence of foveal splaying or inner macular thinning necessitates a thorough examination of the retinal periphery to detect retinal ischemia or neovascularization.

Zhou et al. showed that patients with sickle cell retinopathy especially those with proliferative stages demonstrated asymmetric vascular findings compared to unaffected subjects, with left-eye predominance in disease severity which was compatible with our case. They attributed their findings to anatomical or physiological variables that contribute to the left eye's accelerated disease progression. This could include anatomical differences between the carotid arteries that supply the ophthalmic arteries on the right and left sides. The left common carotid artery arises straight from the aorta, whereas the right common carotid artery arises from the aorta's brachiocephalic branch. Previous investigations have also indicated differences in the responsiveness of the right and left carotid arteries to stresses like hypertension [26, 27].

PSR treatment regimens are not standardized; nonetheless, they all share the common aim of lowering the risk of neovascular lesions progressing to vitreous hemorrhage and/or retinal detachment [28]. Because of the high rate of autoinfarction in SCD induced neovascularization, peripheral retinal nonperfusion and even limited amounts of neovascularization usually do not require any treatment. Nevertheless, laser photocoagulation is recommended when there are extensive neovascularization and vitreous hemorrhage in order to prevent retinal detachment [6]. In a study, the risk of surgical complications in PSR patients was found to be as high as 50% for traction/rhegmatogenous retinal detachment [28]. Therefore, we performed targeted retinal photocoagulation over the peripheral ischemic retina to accelerate the regression of the new vessels.

In conclusion, although SCT appears to be a benign ophthalmic condition in otherwise healthy individuals, it can cause proliferative retinopathy and maculopathy in the presence of other systemic or ocular comorbidities. As pregnancy has considerable effects on the vascular and coagulative systems, it can be one of the potential triggers of retinopathy in SCT patients. Laser photocoagulation therapy should be considered in severe cases of neovascularization and hemorrhage.

## Data Availability

The datasets used in the current study are available upon reasonable request.

## Abbreviations

SCD: Sickle cell disease
SCT: Sickle cell trait
HbA: Hemoglobin A
HbS: Hemoglobin S
HbC: Hemoglobin C
HbA2: Hemoglobin A2
HbF: Hemoglobin F
P-SCR: Proliferative sickle cell retinopathy
OCT: Optical coherence tomography
HIV: Human immunodeficiency virus.

## References

[1] Kato G. J., Piel F. B., Reid C. D. (2018). Sickle cell disease. *Nature Reviews Disease Primers*.

[2] Ware R. E., de Montalembert M., Tshilolo L., Abboud M. R. (2017). Sickle Cell Disease. *Lancet*.

[3] Bonanomi M. T. B. C., Lavezzo M. M. (2013). Sickle cell retinopathy: diagnosis and treatment. *Arquivos Brasileiros de Oftalmologia*.

[4] Abdalla Elsayed M. E. A., Mura M., al Dhibi H. (2019). Sickle cell retinopathy. A focused review. *Graefe's Archive for Clinical and Experimental Ophthalmology*.

[5] Armaly M. F. (1974). Ocular manifestations in sickle cell disease. *Archives of Internal Medicine*.

[6] Goldberg M. F. (1971). Classification and pathogenesis of proliferative sickle retinopathy. *American Journal of Ophthalmology*.

[7] Bachmeier I., Blecha C., Föll J., Wolff D., Jägle H. (2021). *Maculopathy in Sickle Cell Disease*.

[8] Key N. S., Derebail V. K. Sickle-cell trait: novel clinical significance.

[9] Nia J., Lam W. C., Kleinman D. M., Kirby M., Liu E. S., Eng K. T. (2003). Retinopathy in sickle cell trait: does it exist?. *Canadian Journal of Ophthalmology*.

[10] Tsaras G., Owusu-Ansah A., Boateng F. O., Amoateng-Adjepong Y. (2009). Complications associated with sickle cell trait: a brief narrative review. *The American Journal of Medicine*.

[11] Dewundara S., Nassiri N., Kim J. M. (2019). Retinal and choroidal vascular occlusion Following aqueous misdirection syndrome in a patient with sickle cell trait. *RETINAL Cases & Brief Reports*.

[12] Reynolds S. A., Besada E., Winter-Corella C. (2007). Retinopathy in patients with sickle cell trait. *Optometry*.

[13] Nagpal K. C., Asdourian G. K., Patrianakos D. (1977). Proliferative retinopathy in sickle cell trait. Report of seven cases. *Archives of Internal Medicine*.

[14] Camp A. S., Read S. P., Lee R. K. (2019). Peripheral vaso-occlusive events following trauma in patients with sickle cell trait. *Ophthalmic Surgery, Lasers and Imaging Retina*.

[15] Ribeiro J. A. S., Lucena D. R., Lucena L. R., Jorge R. (2009). Proliferative sickle cell retinopathy associated with sickle cell trait and gestational diabetes: case report. *Arquivos Brasileiros de Oftalmologia*.

[16] Jackson H., Bentley C. R., Hingorani M., Atkinson P., Aclimandos W. A., Thompson G. M. (1995). Sickle retinopathy in patients with sickle trait. *Eye*.

[17] Diaw M., Pialoux V., Martin C. (2015). Sickle cell trait worsens oxidative stress, abnormal blood rheology, and vascular dysfunction in type 2 diabetes. *Diabetes Care*.

[18] Wilson S., Ellsworth P., Key N. S. (2020). Pregnancy in sickle cell trait: what we do and don’t know. *British Journal of Haematology*.

[19] Marik P. E., Plante L. A. (2008). Venous thromboembolic disease and pregnancy. *The New England Journal of Medicine*.

[20] Austin H., Lally C., Benson J. M., Whitsett C., Hooper W. C., Key N. S. (2009). Hormonal contraception, sickle cell trait, and risk for venous thromboembolism among African American women. *American Journal of Obstetrics and Gynecology*.

[21] Bhatnagar A., Ghauri A.-J., Hope-Ross M., Lip P. L. (2009). Diabetic retinopathy in pregnancy. *Current Diabetes Reports*.

[22] Condon P. I., Serjeant G. R. (1972). Ocular findings in hemoglobin SC disease in Jamaica. *American Journal of Ophthalmology*.

[23] Lim J. I., Cao D. (2018). Analysis of retinal thinning using spectral-domain optical coherence tomography imaging of sickle cell retinopathy eyes compared to age- and race- matched control eyes. *American Journal of Ophthalmology*.

[24] Fares S., Hajjar S., Romana M. (2021). Sickle cell maculopathy: microstructural analysis using OCTA and identification of genetic, systemic, and biological risk factors. *American Journal of Ophthalmology*.

[25] Serjeant B. E., Mason K. P., Condon P. I. (1984). Blood rheology and proliferative retinopathy in sickle cell-haemoglobin C disease. *The British Journal of Ophthalmology*.

[26] Zhou D. B., Scott A. W., Linz M. O. (2020). Interocular asymmetry of foveal avascular zone morphology and parafoveal capillary density in sickle cell retinopathy. *PLoS One*.

[27] Mehdizadeh M., Lotfi M., Ghoddusi Johari H., Ghassemifar V., Afarid M. (2012). Blood flow parameters of the central retinal and internal carotid arteries in asymmetric diabetic retinopathy. *J. Ophthalmic Vis. Res.*.

[28] Chen R. W. S., Flynn H. W. J., Lee W.-H. (2014). Vitreoretinal management and surgical outcomes in proliferative sickle retinopathy: a case series. *American Journal of Ophthalmology*.

